# Supplementary material for: Homo-composition and hetero-structure nanocomposite Pnma Bi2SeS2 - Pnnm Bi2SeS2 with high thermoelectric performance
Source: Nat Commun. 2021 Dec 10;12:7192. doi: 10.1038/s41467-021-27564-2 (PMC8664806; doi:10.1038/s41467-021-27564-2)
Supplement: Supplementary file 1 — Supplementary information [file 41467_2021_27564_MOESM1_ESM.docx]

**Supporting Information**

**Homo-composition and hetero-structure nanocomposite *Pnma* Bi_2_SeS_2_ - *Pnnm* Bi_2_SeS_2_ with high thermoelectric performance**

**Bushra Jabar^1,#^, Fu Li^1,#,^*, Zhuanghao** **Zheng^1,#^, Adil Mansoor^2^, Yongbin Zhu^3^, Chongbin Liang^1^, Dongwei Ao^1^, Yuexing Chen^1^, Guangxing Liang^1^, Ping Fan^1,^*, and WeishuLiu^3,4,^***

*^1^Shenzhen Key Laboratory of Advanced Thin Films and Applications,* *College of Physics and Optoelectronic Engineering,* *Shenzhen University, Shenzhen, 518060, China.*

*^2^ Faculty of Materials and Manufacturing, Beijing University of Technology, 100 Peenle Yuan, Chaoyang District, Beijing, 100124, China.*

*^3^Department of Materials Science and Engineering, Southern University of Science and Technology, Shenzhen, 518055, China.*

*^4^ Guangdong Provincial Key Laboratory of Functional Oxide Materials and Devices, Southern University of Science and Technology, Shenzhen, 518055, China.*

** Corresponding authors; Emails: lifu@szu.edu.cn, fanping@szu.edu.cn, liuws@sustech.edu.cn*

*^#^ Bushra Jabar, Fu Li and Zhuanghao Zheng contributed equally.*

**1. XRD Rietveld refinement analysis**

Fig. S1 and Fig. S2 depicts the XRD patterns, which indicate that all of the major diffraction peaks from pristine Bi_2_SeS_2_ samples are exclusively indexed as the orthorhombic-structured Bi_2_SeS_2_. However, noticeable and continuous variations in crystal structure upon increasing the doping content in Bi_2_SeS_2_ can be seen. The Rietveld refinement analysis was employed in order to find the details for structural evolution of Bi_2_Se_1-x_Br_x_S_2_ (where x= 0, 0.015, 0.03, 0.06, 0.09, 0.105, 0.12, 0.15, 0.18 and 0.21). In Rietveld refinement, both *Pnma* and *Pnnm* phases were taken into account for all of the XRD refinements, as shown in Fig. S3. Table S1 shows the refined parameters, including the coexistence of phase fractions, unit cell volumes, and lattice parameters for the XRD patterns of Bi_2_Se_1-x_Br_x_S_2_ (where x= 0, 0.015, 0.03, 0.06, 0.09, 0.105, 0.12, 0.15, 0.18 and 0.21). The results demonstrate the doping dependence of the lattice parameters and coexistence of *Pnma* and *Pnnm* phases.





**Fig. S1 Phase structure.** XRD patterns of the *ho*C-*he*S nanocomposite with a normal composition of Bi_2_Se_1-x_Br_x_S_2_ (x= 0, 0.015, 0.03, 0.06, 0.09, 0.105, 0.12, 0.15, 0.18 and 0.21).


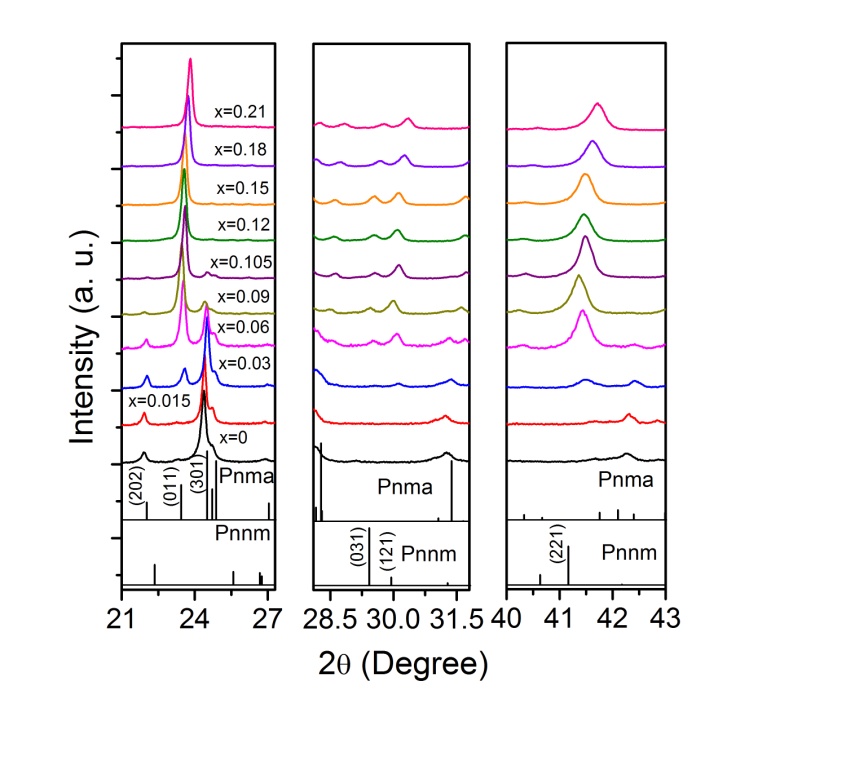


**Fig. S2** **Structural transition of the *ho*C-*he*S nanocomposites.** Some main characteristic peaks of the XRD patterns in Fig. S1 for all the samples Bi_2_Se_1-x_Br_x_S_2_ (x=0-0.21)


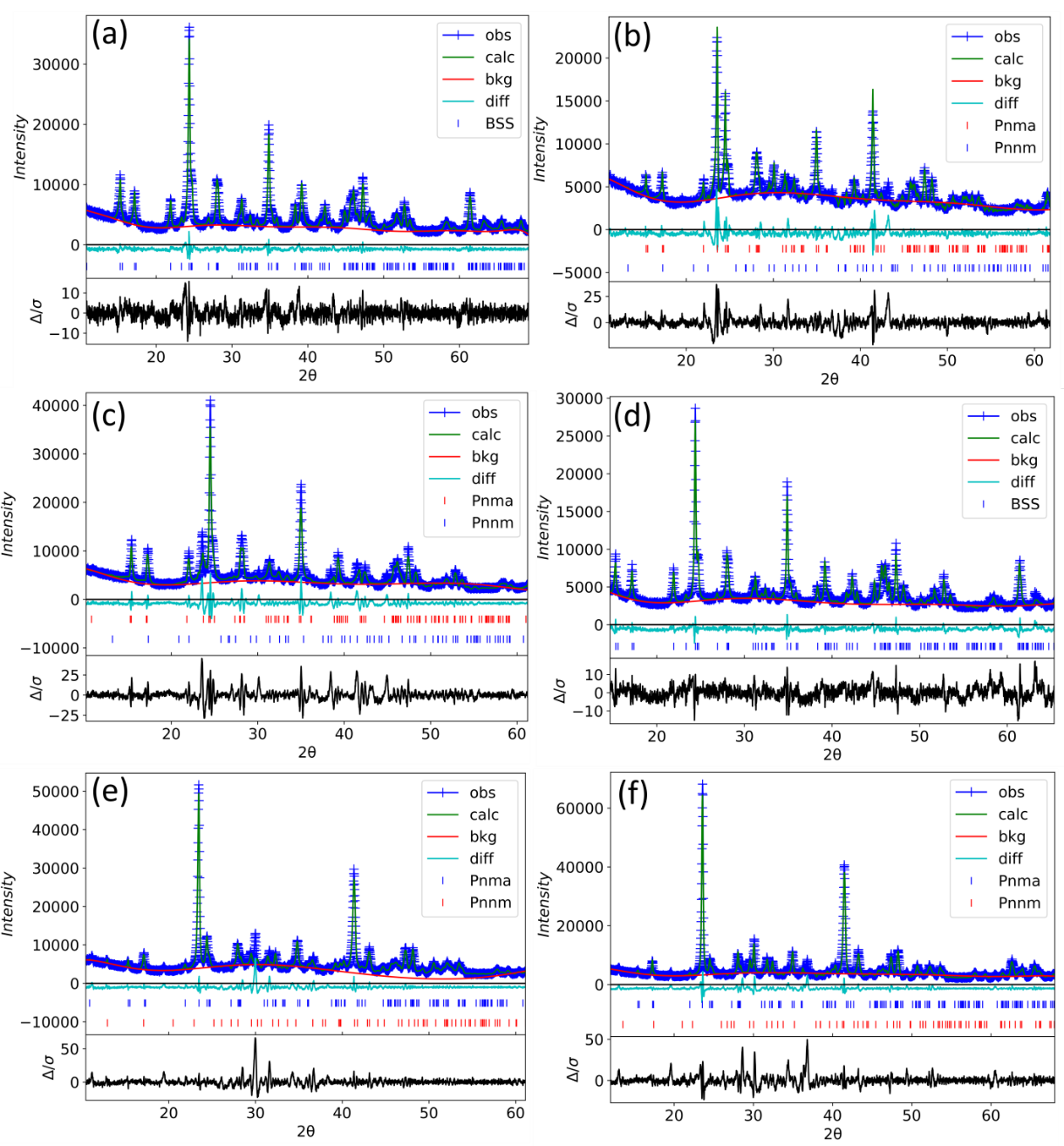


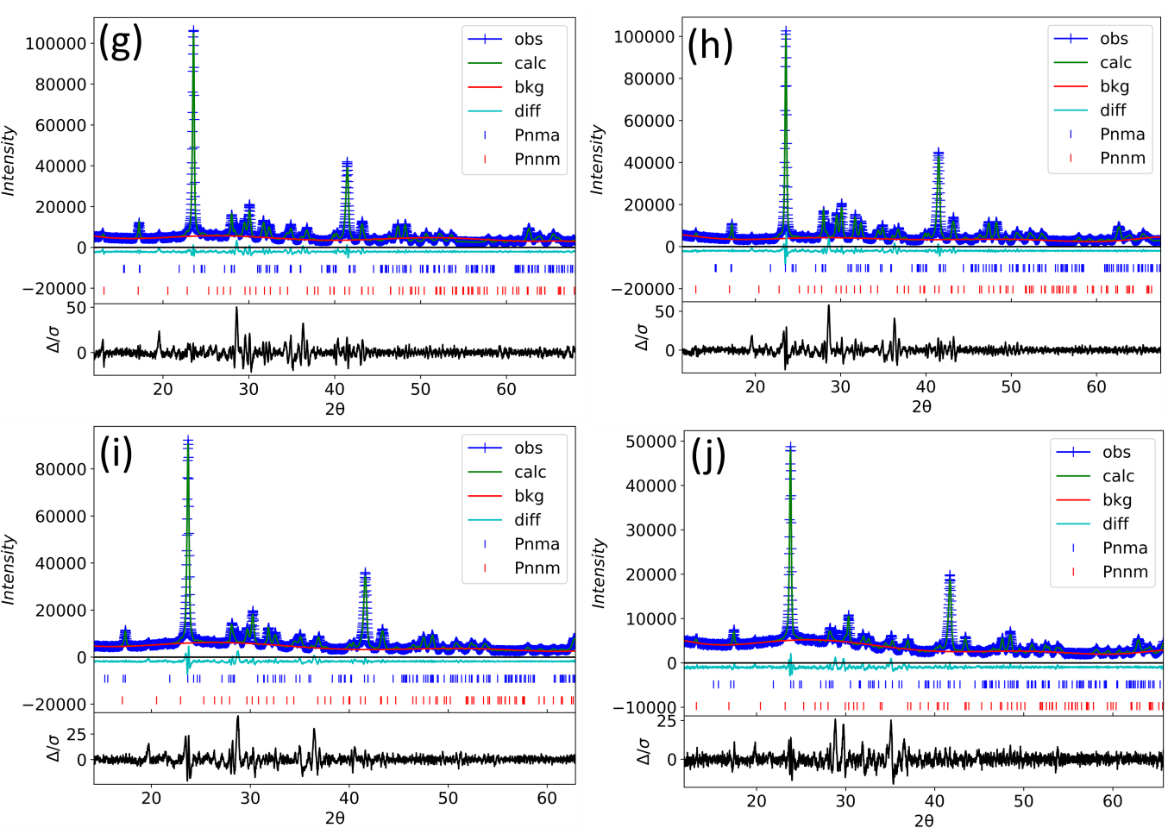


**Fig. S3** **Rietveld refinement analysis.** Rietveld refined analysis from XRD patterns of (a) pure Bi_2_SeS_2_, and the doped Bi_2_Se_1-x_Br_x_S_2_ specimens for x= (b) 0.015, (c) 0.03, (d) 0.06, (e) 0.09, (f) 0.105, (g) 0.12, (h) 0.15, (i) 0.18 and (j) 0.21, where obs, calc, bkg and diff represents observed, calculated, background and residual differences between the curves.

**Table S1.** Rietveld refinement parameters, doping dependent lattice parameters, unit cell volume, and the refinement agreement factors with coexistence phase fractions for all the Bi_2_Se_1-x_Br_x_S_2_ specimens.

| **Sample** |  |  | | ***Pnma*** | |  | |  | |  | | |  |  | | ***Pnnm*** | |  | |  | |  | ***R*_wp_** | |
| --- | --- | --- | --- | --- | --- | --- | --- | --- | --- | --- | --- | --- | --- | --- | --- | --- | --- | --- | --- | --- | --- | --- | --- | --- |
| x | *a*  (Å) | | *b*  (Å) | | *c*  (Å) | | *V*  (Å^3^) | | *f*  (%) | |  | *a*  (Å) | | | *b*  (Å) | | *c* (Å) | | *V*  (Å^3^) | | *f*  (%) | | | *R*_wp_  (%) |
| 0 | 11.525 | | 4.024 | | 11.315 | | 524.75 | | 100 | |  | - | | | - | | - | | - | | - | | | 4.85 |
| 0.015 | 11.499 | | 4.026 | | 11.299 | | 523.09 | | 100 | |  | - | | | - | | - | | - | | - | | | 5.83 |
| 0.03 | 11.399 | | 3.965 | | 11.563 | | 522.65 | | 91.8 | |  | 5.490 | | | 13.462 | | 4.031 | | 297.94 | | 8.2 | | | 9.69 |
| 0.06 | 11.369 | | 3.992 | | 11.495 | | 521.64 | | 82.9 | |  | 5.549 | | | 13.288 | | 4.134 | | 304.83 | | 17.1 | | | 7.89 |
| 0.09 | 11.379 | | 3.999 | | 11.509 | | 523.82 | | 79.9 | |  | 5.584 | | | 13.628 | | 4.036 | | 307.15 | | 20.1 | | | 8.11 |
| 0.105 | 11.402 | | 4.006 | | 11.536 | | 526.92 | | 75.6 | |  | 5.601 | | | 13.587 | | 4.051 | | 308.29 | | 24.4 | | | 8.68 |
| 0.12 | 11.548 | | 4.000 | | 11.483 | | 530.46 | | 71.3 | |  | 5.634 | | | 13.562 | | 4.078 | | 311.53 | | 28.7 | | | 7.53 |
| 0.15 | 11.589 | | 4.000 | | 11.475 | | 531.88 | | 62.4 | |  | 5.655 | | | 13.595 | | 4.067 | | 312.68 | | 37.6 | | | 8.43 |
| 0.18 | 11.659 | | 3.995 | | 11.442 | | 532.97 | | 59.4 | |  | 5.665 | | | 13.556 | | 4.061 | | 311.88 | | 40.6 | | | 6.76 |
| 0.21 | 11.731 | | 3.965 | | 11.323 | | 526.58 | | 44.4 | |  | 5.726 | | | 13.444 | | 4.008 | | 308.58 | | 55.6 | | | 6.04 |


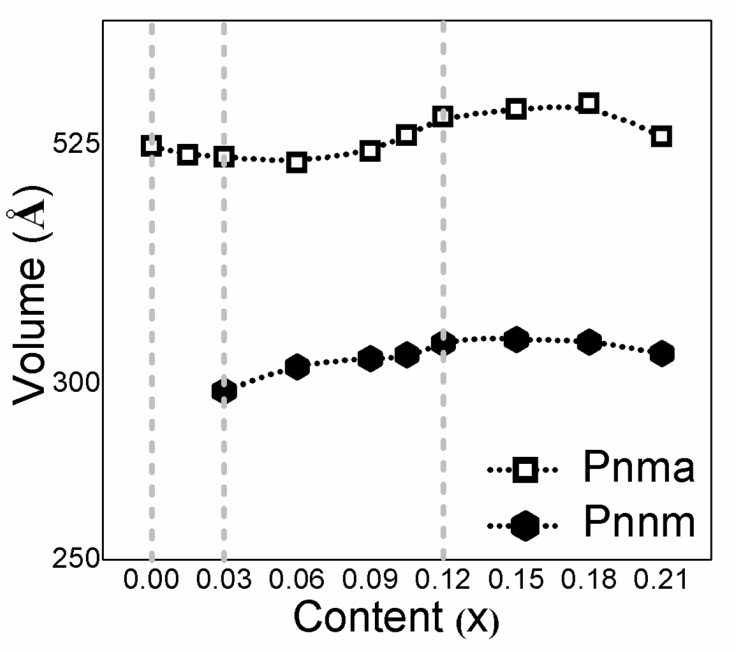


**Fig. S4 Doping induced variation in lattice volume.** Volume derived for *Pnma* and *Pnnm* phases.





**Fig. S5 Disparity in lattice constant parameters.** Lattice constant parameters ratio for *Pnnm* phase Bi_2_SeS_2_.

**2. HRTEM and elemental mapping by HREM-EDS**

**
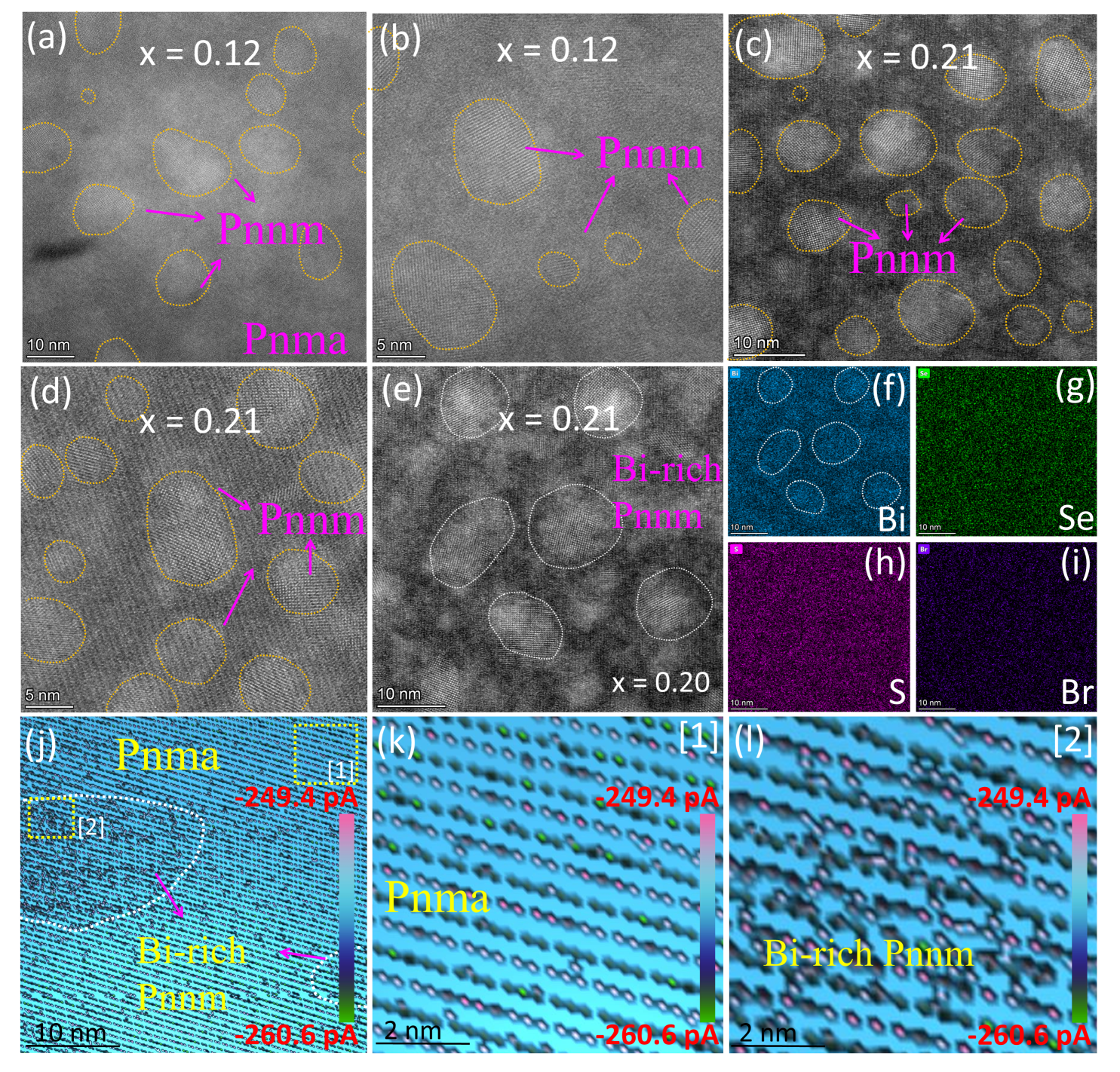
**

**Fig. S6** **Microstructure characteristics.** TEM micrographs for (a) - (b) x = 0.12, and (c) - (d) x = 0.21 for doped Bi_2_Se_1-x_Br_x_S_2_ specimens. (e) - (i) TEM elemental mapping for Bi, S, Se and Br corresponding to (f), (g), (h) and (i), respectively.





**Fig. S7 Doping dependent disparity in the bond distances.** Bond length values for different bonds, where dotted lines represent theoretically derived lattice bond lengths.


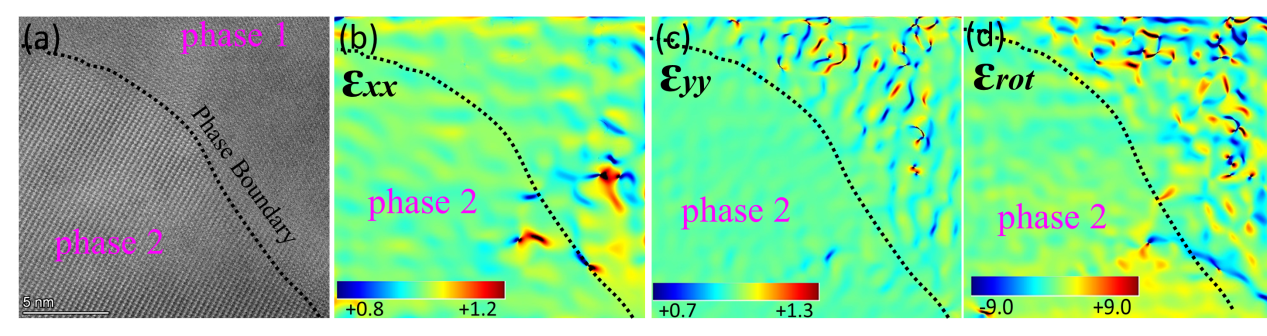


**Fig. S8 Geometric phase analysis (GPA) of corresponding HRTEM images from Fig. 4 (c) in the main text**. (a) HRTEM micrograph; (b) - (d) Corresponding strain maps along the ɛ*_xx_* , ɛ*_yy_* and ɛ*_rot_* for (a).

**3. Thermoelectric properties**

As we know, Seebeck coefficient (*S*) is not only dependent on carrier concentration but also on other physical parameters. In relaxation time approximation, µ(E) = eτ(E)/m^∗^ (here, τ(E) and m^∗^ are relaxation time and effective mass of carrier, respectively) and energy filtering effect is realted to scattering parameter with the relation of τ(E)=τ_0_E^λ-1/2^ (where E is energy of carriers and τ_0_ is energy dependent constant). According to Mott formula, one can simply write the above expression as:

$$S=S(n, m_{d}^{*}, , T) (S1)$$

where T is temperature, *n* is carrier concentration, $m_{d}^{*}$is the density of states effective mass, and λ is the scattering parameter. From formula (S1), one can see clearly that: as $m_{d}^{*}$, λ and T are kept as constants, *S* should decrease with increasing carrier concentration *n*, as predicted by Pisarenko relation. However, as $m_{d}^{*}$ or λ changes (increases), *S* values will (enhance as compared with Bi_2_SeS_2_) deviate from Pisarenko relation (as shown in Fig. S9(b)). Therefore, (at a given temperature) whether *S* value deviates from Pisarenko relation is a criterion for the change of λ (λ does not change significantly here) or/and $m_{d}^{*}$(in our doped system). In order to determine whether the energy-dependent carrier scattering mechanism works and contributes to the enhancement of *S* in this system, density of state effective mass (*m*^*^_d_) can be obtained from the single parabolic band model, as following,^1-3^

$$S=\frac{k_{B}}{e}\left[ \frac{\left( \lambda+2 \right)F_{\lambda+1}\left( \xi_{F} \right)}{\left( \lambda+1 \right)F_{\lambda}\left( \xi_{F} \right)}-\xi_{F} \right] (S2)$$

$$m_{d}^{*}=\frac{h^{2}}{2k_{B}T}{(\frac{n}{4\pi F_{1/2}(\xi_{F})})}^{2/3} (S3)$$

Where $F_{j}\left( \xi_{F} \right)=\int_{0}^{\infty} \frac{x^{j}}{1+e^{x-\xi_{F}}}\mathrm{dx}$(S4) (S4)

is the Fermi integral of order j, ξ_F_ is reduced Fermi level E_f_/k_B_T and *h* is Plank constant. By using the data of *S* and *n*, and assuming acoustic phonon scattering is prominent (i.e. λ =0) in Bi_2_SeS_2_ matrix (*x*=0), one can obtain Pisarenko relation (at 300 K) for Bi_2_SeS_2_ sample (the solid line in Fig. S9(b)) via formulae (S2)-(S4). It is found that the *S* values for the samples with x larger than 0.06 deviate the Pisarenko relation. In order to determine whether the density of state effective mass (*m*^*^_d_) works and contributes to the *S*, *m*^*^_d_ are obtained using the equations (S2)-(S4), assuming the current composition is single uniform phase. Fig. S9(c) shows the variation of density of state effective mass with doping content x. The variations in *m*_d_* values at different Br content reveal that the band structure is dependent on the Br doping under inter-orthorhombic phase transformation.

The Femi energy at room temperature can also be estimated via *S* by using the equations (S2)-(S4). In addition, according to the Goldsmid-Sharp relation, *S*_max_=*E_g_*/2*eT*_max_, where *e* is the elementary charge, the Seebeck band gap *E*_g_ can be estimated by the maxima in *S*(T) with the coordinates (*T*_max_, *S*_max_).^4^ Table S2 shows the Seebeck band gap and Fermi energy for all the samples. It can be found that the band gap has been roughly reduced after Br doping. As for the Femi energy, it first increases after doping and then reduces with further increasing the doped content x, indicating the change of the electronic density of states. It should be noted that Eq. (S2)-(S3) above are derived from the assumption of single uniform phase. In our case, we have two phase, which may result into the physical meaning of *E_g_* is not very clear.


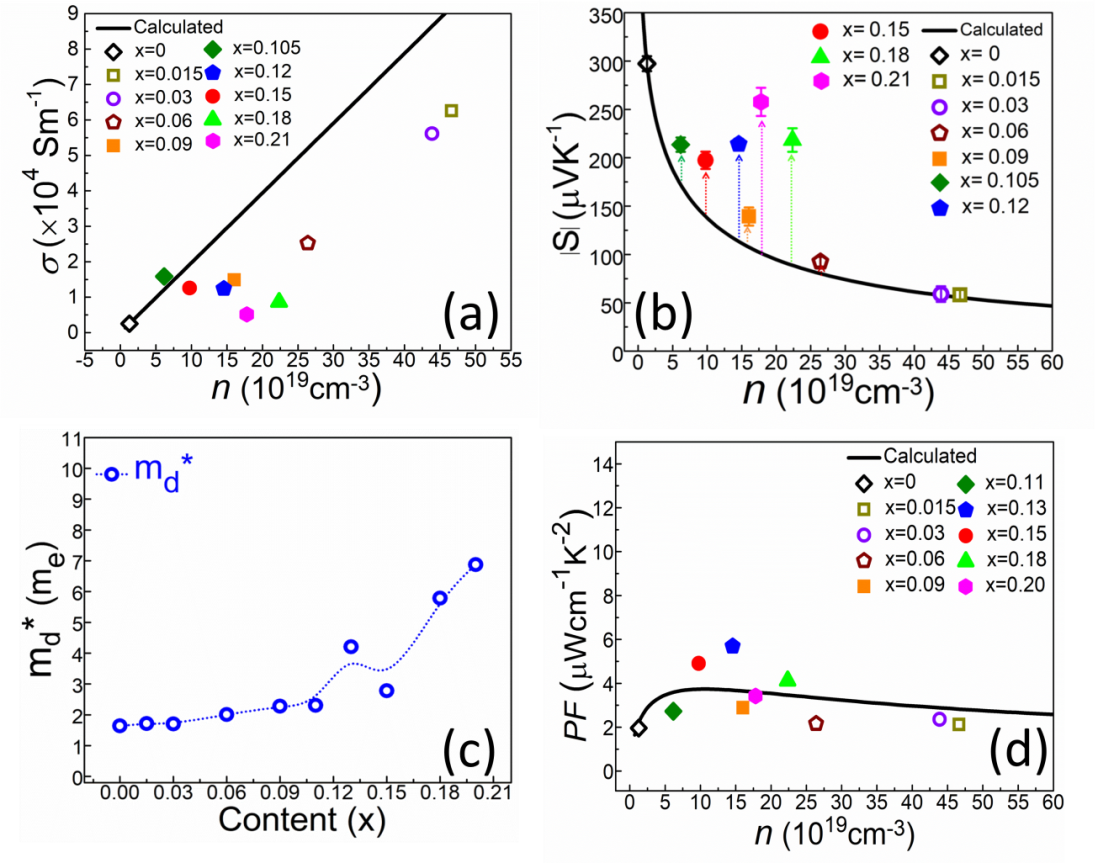


**Fig. S9 Electrical transport properties for the fabricated samples.** (a) Electrical conductivity as a function of carrier concentration (*n*), (b) Seebeck coefficient (*S*) as a function of carrier concentration (*n*), (c) effective mass and (d) variation of power factor (*PF*) with carrier concentration *n* (at 300 K).

**Table S2** Seebeck coefficient (*S*), estimated Seebeck band gap (*Eg*) and Fermi energy (*EF*) at room temperature for all the samples Bi_2_Se_1-x_Br_x_S_2_ (x: 0-0.21).

| x | 0 | 0.015 | 0.03 | 0.06 | 0.09 | 0.105 | 0.12 | 0.15 | 0.18 | 0.21 |
| --- | --- | --- | --- | --- | --- | --- | --- | --- | --- | --- |
| *S*(μV/K) | 297.2 | 58.2 | 58.9 | 92.5 | 139.3 | 231.4 | 214.1 | 197.3 | 218.3 | 257.8 |
| *Eg*(eV) | 0.47 | 0.27 | 0.26 | 0.28 | 0.32 | 0.24 | 0.2 | 0.21 | 0.21 | 0.23 |
| *EF*(eV) | -0.035 | 0.124 | 0.122 | 0.071 | 0.033 | -0.0036 | -0.0042 | 0.0031 | -0.0057 | -0.021 |

**4. Formation energy**

The formation energy of Bi_2_SeS_2_ compound,$E_{for}^{Bi2SeS2}$, is calculated according to Equation S5^5,6^:

$$E_{for}^{Bi2SeS2}=E_{tot}\left( \mathrm{Bi}_{2}\mathrm{Se}S_{2} \right)-2\mu_{\mathrm{Bi}}- \mu_{\mathrm{Se}}- 2\mu_{S} (S5)$$

where E_tot_(Bi_2_SeS_2_) is the total energy of the Bi_2_SeS_2_ compound, and μ_Bi_, μ_Se_ and μ_S_ are the chemical potentials of Bi, Se and S, respectively. The chemical potentials are equal to the DFT total energies of their ground states. The formation energy of a Br substituting a Se$\Delta E_{for}\left( \mathrm{Br}_{\mathrm{Se}} \right)$(and S$\Delta E_{for}\left( \mathrm{Br}_{S} \right)$), is given by;

$$\Delta E_{for}\left( \mathrm{Br}_{\mathrm{Se}} \right)={E_{tot}\left( \mathrm{Br}_{\mathrm{Se}} \right)-E}_{tot}\left( \mathrm{Bi}_{2}\mathrm{Se}S_{2} \right)+\mu_{\mathrm{Se}}- \mu_{\mathrm{Br}} (S6)$$

Where $E_{tot}\left( \mathrm{Br}_{\mathrm{Se}} \right)$, $E_{tot}\left( \mathrm{Bi}_{2}\mathrm{Se}S_{2} \right)$ are the DFT total energies of the doped and pristine cell, respectively, and $\mu_{\mathrm{Se}}$and $\mu_{\mathrm{Br}}$are the chemical potential of Se and Br.


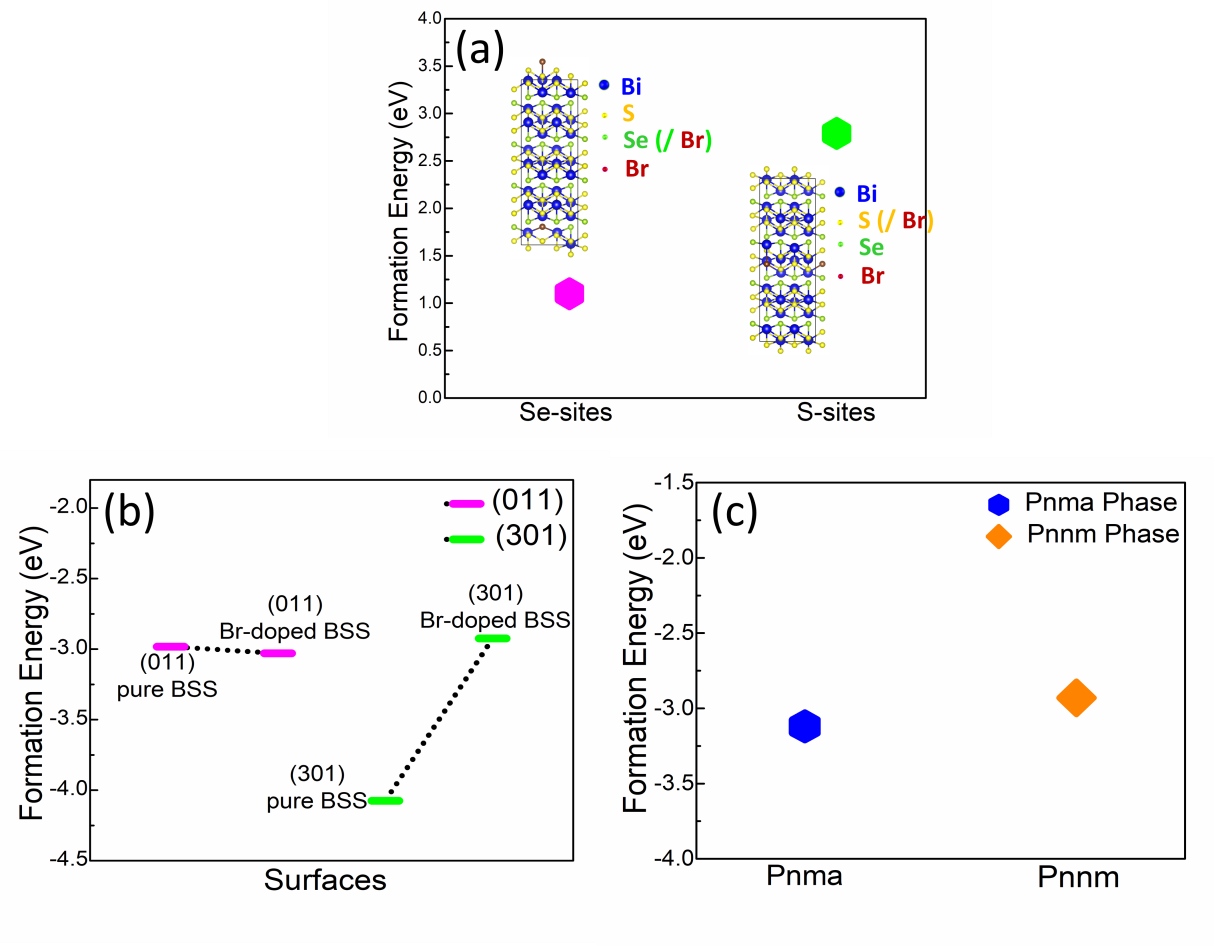


**Fig. S10 Formation energy calculation.** (a) Formation energy values for Br doping at Se and S-sites, (b) Calculated formation energies for Bi_2_SeS_2_ along plane (011) & (301), (c) Calculated formation energies for *Pnma* and *Pnnm* phases.

**5. Thermal conductivity and *ZT***

The total thermal conductivity (*к*) for all the samples is shown in Fig. S11 (a). The electronic thermal conductivity (*к_e_*) was calculated from the equation of *к_e_*=*LσT*, where *σ* is the electrical conductivity, *T* is the temperature, *L* is the Lorenz number obtained by applying the calculated reduced Fermi energy *η* and scattering parameter *r*, ranging from 1.5×10^-8^ to 2.0×10^-8^ V^2^/K^2^. The temperature-dependent *к_e_* of all the samples Bi_2_Se_1-x_Br_x_S_2_ is shown in Fig. S11 (b), which presents good consistency with the electrical conductivity *σ*. Compared with the undoped sample, the *к_e_* increases firstly after Br doping due to the enhanced electrical conductivity and holds ~36% of *к* for the doped sample with x=0.015. And then it reduces because of the decrease of the electrical conductivity and contributes ~18% of *к* for the sample with doped content x=0.09. Thus, it suggests that the lattice thermal conductivity dominates the total thermal conductivity.

For a wide-gap semiconductor at elevated temperature or narrow-gap semiconductor at ordinary temperature, both electrons and holes contributing to the transport. In these cases, the bipolar thermal conductivity *κ*_b_ should be considered. Thus, the total thermal conductivity (*κ*) can be written in the form: *κ*=*κ*_L_+*κ*_e_+*κ*_b_, where *κ*_L_ is the lattice thermal conductivity and *κ*_e_ is the electronic thermal conductivity. In order to clarify the contribution of *κ*_b_ at high temperatures in the present study, the *κ*_b_ is separated from the *κ* according to the method proposed by Kitagawa *et al.*^7^ The difference, *κ*_L_+*κ*_b_ as a function of *T*^-1^ for the sample Bi_2_Se_1-x_Br_x_S_2_ is shown in Fig. S11(c). Since the acoustic phonon scattering is predominant at low temperature, the *κ*_L_+*κ*_b_ equals to *κ*_L_, which is proportional to *T*^-1^. With increasing the temperature, *κ*_L_+*κ*_b_ started to gradually deviate from such a linear relationship between *κ*_L_ and *T*^-1^. This implies that the bipolar diffusion starts to contribute to the thermal conductivity. The *κ*_L_ at high temperature was estimated by extrapolating the linear relationship between *κ*_L_ and *T*^-1^, as indicated by the dotted line in Fig. S11(c). Hence, *κ*_b_ at high temperature should be equal to *κ*-*κ*_L_-*κ*_e_. Fig. S11(d) shows the temperature dependence of the *κ*_b_ for some representative samples. It can be seen that with increasing Br content x from 0 to 0.12, *κ*_b_ shows decreasing trend mainly due to the trapping of minority carriers within the intensive nanoscale regions inside the doped samples. However, *κ*_b_ increases with further increasing the doping x ≥ 0.15 which can be ascribed to the extra carrier regenation/recombinaion with the unconfined minority carriers due to agglomerated nanophase or reduced nanoscale regions.

**
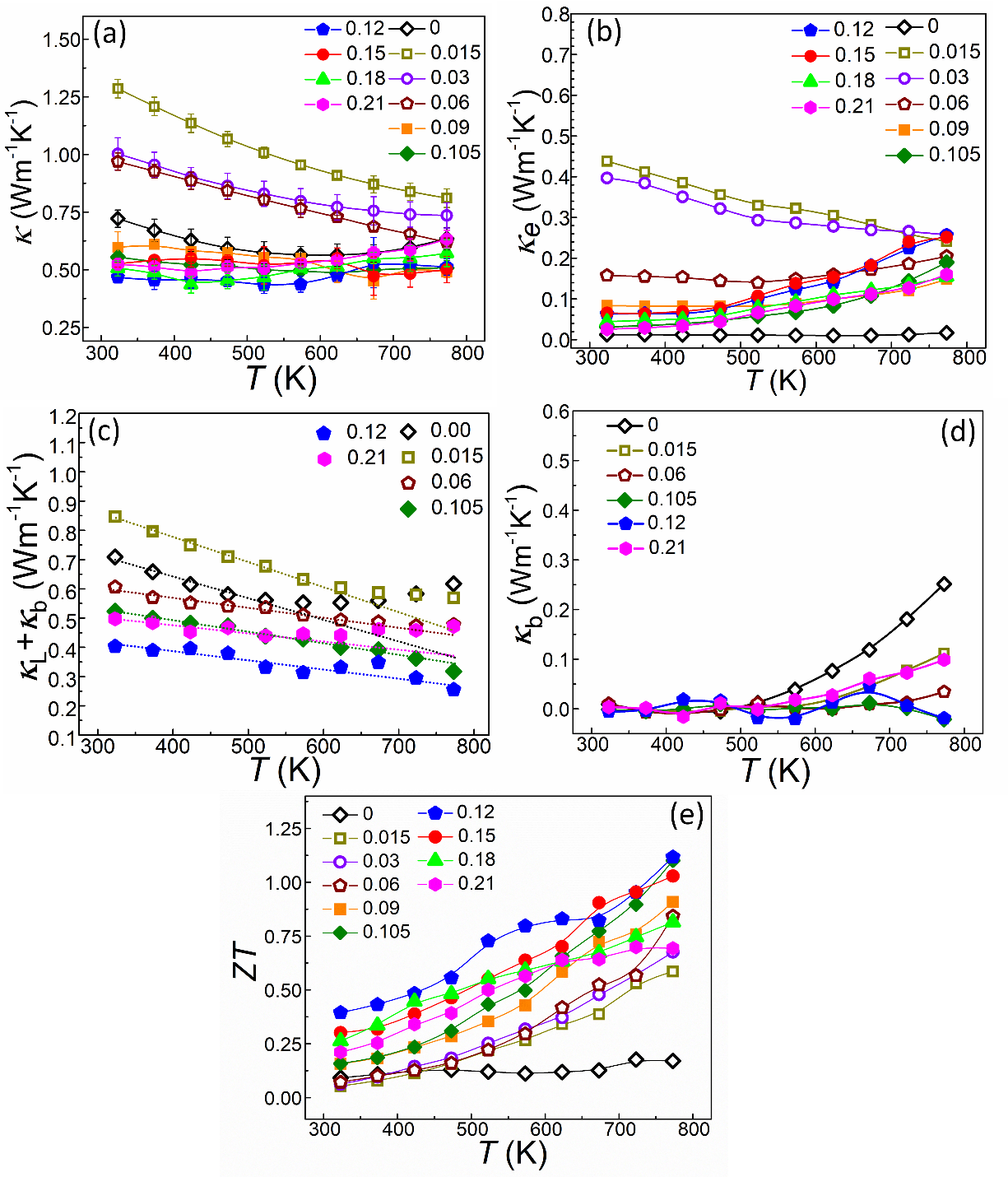
**

**Fig. S11 Thermal transport properties and *ZT* values.** (a) Temperature dependence of the total thermal conductivity (*κ*). (b) Temperature dependence of the electronic thermal conductivity (*κ_e_*). (c) and (d) Temperature dependence of the lattice thermal conductivity (*κ_L_*) and bipolar thermal conductivity (*κ_b_*) for some representative samples Bi_2_Se_1-x_Br_x_S_2_. The dotted line in (c) is linearly fitting to the lattice thermal conductivity (*κ*_L_) at low temperature. (e) *ZT* values for Bi_2_Se_1-x_Br_x_S_2_ (x=0, 0.015, 0.03, 0.06, 0.09, 0.105, 0.12, 0.15, 0.18, and 0.21).

**6. Lattice thermal conductivity calculations**

In present work, the decrease of *κ*_L_ upon doping is explained by Debye-Callaway model. The total phonon relaxation time τ_T_ for phonons in doped Bi_2_SeS_2_ samples can be expressed as:^1^

$\tau_{T}^{-1}= \tau_{PD}^{-1}+{\tau_{U}^{-1}+\tau}_{NI}^{-1}+\tau_{EP}^{-1}$(S7)

where $\tau_{PD}^{-1},\tau_{U}^{-1}, \tau_{EP}^{-1}, \mathrm{and}\tau_{NI}^{-1}$ are relaxation time related to phonon scattering from point defects or impurities, Umklapp processes, electron-phonon scattering and phonon scattering from phase boundaries between *Pnma* Bi_2_SeS_2_ and *Pnnm* Bi_2_SeS_2_ phases, respectively. As a result, τ_T_ (the total phonon relaxation time) for doped system reduces as compared to τ_T_ for undoped Bi_2_SeS_2_ due to the existence of$\tau_{PD}^{-1},\tau_{U}^{-1}, \tau_{EP}^{-1}\mathrm{and}\tau_{NI}^{-1}$in doped matrix. Then, according to Debye-Callaway model,^8,9^

$$\kappa_{L}=\frac{4\pi{k_{B}}^{4}T^{3}}{\nu h^{3}}\int_{0}^{\frac{\theta_{D}}{T}} \tau_{T}\frac{z^{4}exp(z)}{[exp \left( z \right)-{1]}^{2}}\mathrm{dz} (S8)$$

Here, θ_D_, and z stand for Debye temperature and reduced phonon frequency, respectively. In fact, in Bi_2_Se_1-x_Br_x_S_2_ system, the dominant phonon-scattering mechanisms involves scattering processes from point defects/alloy elements, phonon-phonon Umklapp-scattering, phase boundaries between *Pnma* Bi_2_SeS_2_ and *Pnnm* Bi_2_SeS_2_ phases, electron-phonon interaction. Generally, in un-doped Bi_2_SeS_2_, points defects including alloy elements and impurities with size about ~0 origins to initiate only intense scattering of short-wavelength phonons or brillion zone edge phonons without effective scattering of mid- and long-wavelength phonons, leading no significant reduction in *κ*_L_. However, scattering cross section ϕ mainly depends on size of scattering particles (b) and phonon wavelength (*l*), which can be stated by a relation; ϕ ~ b^6^/*l*^4^ (or b^6^ω^4^). Thus, in the heavily doped system, scattering cross section ϕ increases with the presence of nanophase sizes about ~10 - 100 nm and causes to initiate the extensive scattering of mid- and long-wavelength phonons, leading to sharp reduction of *κ*_L_.^6^ Hereafter, intrusive phonon scattering for impurity/point defect phonon scattering, phonon-phonon Umklapp scattering and electron-phonon scattering inside the system from point defects/alloy elements owing to their Rayleigh scattering character can be expressed as;

$$\tau_{PD}^{-1}=\frac{V \Gamma}{4\pi\nu^{3}}\omega^{4}\propto A\omega^{4} (S9)$$

$$\tau_{U}^{-1}=\frac{{{\hbar\gamma}^{2}\omega}^{2}T}{M\nu^{2}\theta_{D}}exp\left( -\frac{\theta_{D}}{3T} \right)\propto B\omega^{2} (S10)$$

$$\tau_{EP}^{-1}= {\beta\tau}_{U}^{-1}=\beta\frac{{{\hbar\gamma}^{2}\omega}^{2}T}{M\nu^{2}\theta_{D}}exp\left( -\frac{\theta_{D}}{3T} \right)\propto C\omega^{2} (S11)$$

Then, it can simplify as,

$$\tau_{T}^{-1}=A\omega^{4}+B\omega^{2}T\exp\left( -\frac{\theta_{D}}{3T} \right)+C\omega^{2}+v/L (S12)$$

here, ω is the phonon frequency and L is the average spatial distance among the nanophase/nanoparticles in Bi_2_SeS_2_, while ν/L corresponds to scattering from the phase boundaries. Hence, by substituting formula (S9) - (S12) (but excluding term v/L for the undoped Bi_2_SeS_2_) for $\tau$_T_ in Debye model (formula (S8)) and by fitting experimental data of κ_L_ for Bi_2_SeS_2_ to formula (S8), one can obtain parameters A, B and C in formula (S12) for the undoped sample. Then, by using the obtained A, B and C parameters, one can calculate κ_L_ for the typical Br doped samples with *x ≥* 0.015 through formula (S8) and formula (S12). The calculated results are given (the dotted line) in Fig. 7(b) in the main text. In this calculation process, phonon-phonon Umklapp scattering ${(\tau}_{U}^{-1})$ and electron-phonon scattering ${(\tau}_{EP}^{-1})$are kept as constant which are same as those for the undoped sample, because they are independent on induced defects and phase boundaries in present doped samples. The phonon scattering from impurity/point defect ${(\tau}_{PD}^{-1})$and phase boundaries$(v/L)$ were mainly taken into account to find the effect of Br doping on lattice thermal conductivty. In this work, the values of ν=2775 ms^-1^ and θ_D_ =283 K were taken from literatures.^5-7^

**Table S3. Parameters for the calculation of Callaway model**

| **x** | **A** (10^-40^ s^3^) | **B** (10^-18^ s/K) | **C** (10^-16^ s) | **L** (nm) |
| --- | --- | --- | --- | --- |
| 0 | 7.35 | 8.95 | 8.98 | ------ |
| 0.015 | 3.48 | 8.95 | 8.98 | 900 |
| 0.03 | 4.34 | 8.95 | 8.98 | 850 |
| 0.06 | 5.35 | 8.95 | 8.98 | 630 |
| 0.09 | 6.34 | 8.95 | 8.98 | 100 |
| 0.105 | 9.37 | 8.95 | 8.98 | 80 |
| 0.12 | 10.4 | 8.95 | 8.98 | 30 |
| 0.15 | 8.36 | 8.95 | 8.98 | 77 |
| 0.18 | 7.93 | 8.95 | 8.98 | 80 |
| 0.21 | 7.49 | 8.95 | 8.98 | 110 |

**7. Repeatability and thermal stability for Bi_2_Se_1-x_Br_x_S_2_ (x=0.12)**

**
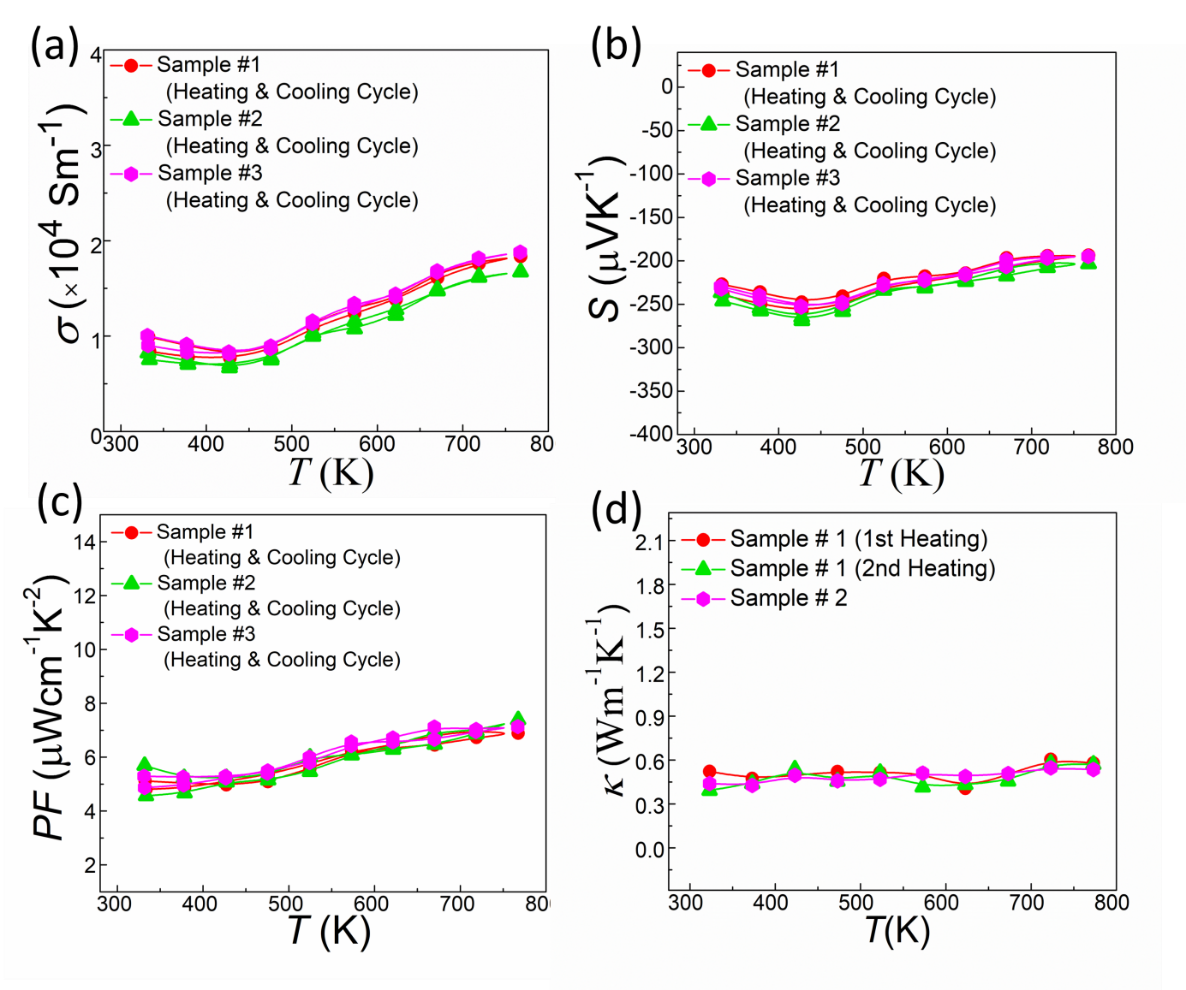
**

**Fig. S12 Repeatability and thermal stability of Bi_2_Se_1-x_Br_x_S_2_ (x=0.12) samples.** (a) electrical conductivity (*σ*), (b) Seebeck coefficient (*S*), (c) power factor (*PF*), and (d) thermal conductivity (*κ*).

**8. Comparison of the thermoelectric properties**

**Table S4** Thermoelectric properties, including electrical conductivity (*σ*), Seebeck coefficient (*S*), thermal conductivity (*κ*) and *ZT*_max_ value at the optimum temperature (*T*_max_) of the present Bi_2_Se_1-x_Br_x_S_2_ (*x*=0.12) and other reported Bi_2_S_3_ related compounds.

| Sample | *σ*(Scm^-1^) | *S*(μVK^-1^) | *κ*(Wm^-1^K^-1^) | *ZT*_max_ | *T*_max_(K) |
| --- | --- | --- | --- | --- | --- |
| Bi_2_Se_1-x_Br_x_S_2_ (*x*=0.12) | ~210 | 187 | 0.51 | 1.12 | 773 (this work) |
| Bi_2_S_3_+1.5mol%PbBr_3_ | ~90 | 260 | 0.50 | ~0.8 | 673^10^ |
| Bi_2-x_Sn_x_S_3_ (x= 0.015) | ~65 | ~ -190 | ~0.22 | 0.67 | 673^11^ |
| Cu_0.01_Bi_2_S_3_ | ~67 | ~ -250 | ~0.5 | 0.62 | 723^12^ |
| Cu_0.02_Bi_2_SeS_2_ | 146 | ~ -200 | ~0.36 | 0.75 | 723^13^ |
| Bi_2_S_3_+0.5mol% CuCl_2_ | ~110 | ~ -220 | ~0.55 | ~0.8 | 760^14^ |
| Bi_2_S_3_+0.5mol% BiCl_3_ | 107 | -233 | ~0.80 | 0.6 | 760^15^ |
| Bi_2_S_3_/Bi | ~10 | ~ -400 | ~0.36 | 0.36 | 623^16^ |
| Bi_2_S_3_ | ~200 | ~ -150 | ~0.65 | 0.5 | 723^17^ |
| Bi_2_S_3_+1.0 mol% ZnO | ~75 | ~ -250 | ~0.47 | 0.66 | 675^18^ |
| Bi_2_S_3_+1.0 mol% BiI_3_ | 22 | ~ -370 | 0.42 | 0.58 | 773^19^ |
| Bi_2_SeS_2_+3mol% CuBr | ~140 | ~ -200 | ~0.55 | 0.71 | 723^20^ |
| Bi_2_S_3_+1 mol% InCl_3_ | 62 | -244 | 0.42 | 0.57 | 673^21^ |
| Bi_2_SeS_2_+*x*CuI (*x*=0.02) | ~129 | 220 | 0.47 | 1.04 | 773^22^ |





**Fig. S13 Comparison of *ZT*_max_ value at the optimum temperature (*T*_max_).** *ZT*_max_ values of the present Bi_2_Se_1-x_Br_x_S_2_ (x=0.12) and other reported n-type sulfide compounds in the last two years. ^12,22-32^

**9. Hall resistance**

Fig. S14 shows the variation of the Hall resistance (*R_H_=V_h_/I*) with magnetic field (B). It can be found that *R*_H_ shows a linear behavior with the magnetic field for all the samples, indicating the as-fabricated Bi_2_SeS_2_ nonacomposition can be assumed as uniform phase using a single parabolic band model for the calculation. Therefore, the Pisarenko relation as exhibited in Fig. S9, which was obtained by assuming single parabolic band model, should be reasonable in the present study.


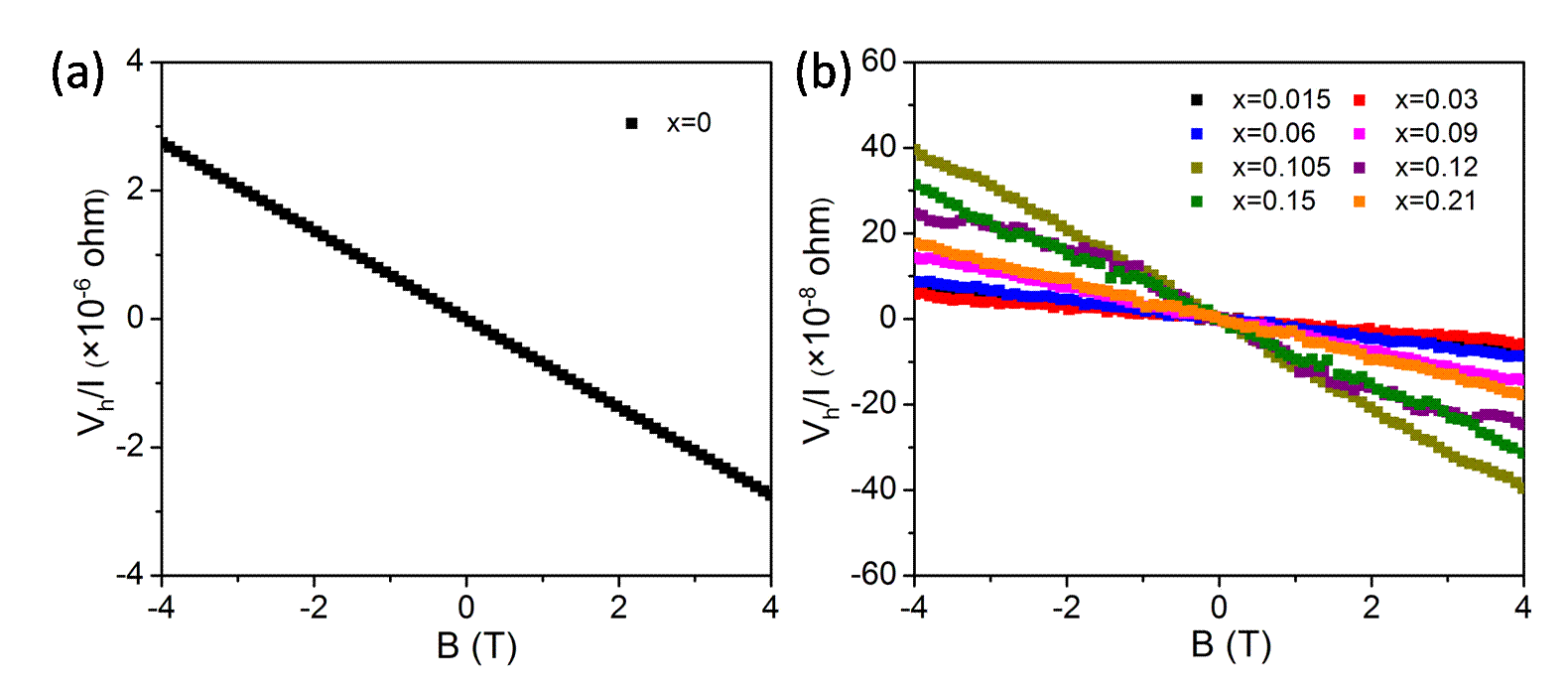


**Fig. S14 Variation of Hall resistance (*R_H_=V_h_/I*) with magnetic field (*B*).** (a) Br-free sample Bi_2_SeS_2_, (b) Br doped samples Bi2Se1-xBrxS2.

**References**

1. Ming, H. et al*.* Boosting thermoelectric performance of Cu_2_SnSe_3_ via comprehensive band structure regulation and intensified phonon scattering by multidimensional defects. *ACS Nano* **15**, 10532-10541 (2021).
2. Jabar B. et al. Achieving high thermoelectric performance through constructing coherent interfaces and building interface potential barriers in n-type Bi_2_Te_3_/Bi_2_Te_2.7_Se_0.3_ nanocomposites. *J. Mater. Chem. A* **7**, 19120-19129 (2019).
3. Jabar B. et al. Enhanced power factor and thermoelectric performance for n-type Bi_2_Te_2.7_Se_0.3_ based composites incorporated with 3D topological insulator nanoinclusions. *Nano Energy* **80**, 105512 (2021).

4. Ciesielski, K. et al. Thermoelectric performance of the half heusler phase RNiSb (R=Sc,Dy Er, Tm, Lu): High mobility between majority and minority charge carriers, *Phys. Rev. Appl.*, **14**, 054046 (2020).

5. Do, D. T. & Mahanti, S. D. Theoretical study of defects Cu_3_SbSe_4_: Search for optimum dopants for enhancing thermoelectric properties. *J. Alloys Compd.* **625**, 346-354(2015)

6. Emery, A. A. & Wolverton, C. High-throughput DFT calculations of formation energy, stability and oxygen vacancy formation energy of ABO_3_ perovskites. *Scientific Data* **4**, 170153 (2017).

7. Kitagawa, H. et al. Temperature dependence of thermoelectric properties of Ni-doped CoSb_3_. *J. Phys. Chem. Solids* **66**, 1635 (2005).

8. Rowe, D. M. *Thermoelectrics handbook: macro to nano*. (CRC press, 2018).

9. Majumdar, A. Thermoelectricity in Semiconductor Nanostructures. *Science* **303**, 777-778 (2004).

10. Guo, J. et al, High thermoelectric properties realized in earth-abundant Bi_2_S_3_ bulk via carrier modulation and muti-nano-precipitates synergy. *Nano Energy* **78**, 105227(2020).

11. Guo, Y., Du, X. L., Wang, Y. L. & Yuan, Z. H. Simultaneous enhanced performance of electrical conductivity and Seebeck coefficient in Bi_2-x_Sn_x_S_3_ by solvothermal and microwave sintering. *J. Alloys Compd.* **717**, 177-182 (2017).

12. Yang, J. et al. Thermoelectric properties of n-type Cu_x_Bi_2_S_3_ materials fabricated by plasma activated sintering. *J. Alloys Compd.* **780**, 35-40 (2019).

13. Li, L. et al. Thermoelectric property studies on Cu_x_Bi_2_SeS_2_ with nano-scale precipitates Bi_2_S_3_. *Nano Energy* **12,** 447-456 (2015).

14. Ji, W. T. et al. Boosting the thermoelectric performance of n-type Bi_2_S_3_ by hierarchical structure manipulation and carrier density optimization. *Nano Energy* 106171 (2021).

15. Biswas, K., Zhao, L. -D & Kanatzidis, M. G. Tellurium‐free thermoelectric: the anisotropic n-type semiconductor Bi_2_S_3_. *Adv. Energy Mater.* **2**, 634-638 (2012).

16. Ge, Z. H. et al. Highly enhanced thermoelectric properties of Bi/Bi_2_S_3_ nanocomposites. *ACS Appl. Mater. Inter.***29**, 4828-4834 (2017).

17. Liu, W. S. et al. Bi_2_S_3_ nanonetwork as precursor for improved thermoelectric performance. *Nano Energy* **4**, 113-122 (2014).

18. Du, X. et al. Enhanced thermoelectric performance of n-type Bi_2_S_3_ with added ZnO for power generation. *RSC Adv.* **5**, 31004-31009 (2015).

19. Yang, J. et al. Enhanced the thermoelectric properties of n-type Bi_2_S_3_ polycrystalline by iodine doping. *J. Alloys Compd.* **728**, 351-356 (2017).

20. Ruan, M., Li, F., Chen, Y. X., Zheng, Z. H. & Fan, P. Te-free compound Bi_2_SeS_2_ as a promising mid-temperature thermoelectric material. *J. Alloys Compd.* **849**, 156677 (2020).

21. Guo, J., Ge, Z. H., Qian, F., Lu, D. H. & Feng, J. Achieving high thermoelectric properties of Bi_2_S_3_ via InCl_3_ doping. *J. Mater. Sci.* **55**, 263-273 (2020).

22. Li, F., et al. High thermoelectric properties achieved in environmentally friendly sulfide compound Bi_2_SeS_2_ by nanoenginnering. *Nano Energy* **88**, 106273 (2020).

23. Bourgès, C. et al*.* Tailoring the thermoelectric and structural properties of Cu–Sn based thiospinel compounds [CuM_1+ x_Sn_1− x_S_4_ (M= Ti, V, Cr, Co)]. *J Mater. Chem. C* **8**, 16368-16383 (2020).

24. Labégorre, J.-B. et al. XBi_4_S_7_ (X = Mn, Fe): New cost-efficient layered n-type thermoelectric sulfides with ultralow thermal conductivity. *Adv. Funct. Mater.* **29**, 1904112 (2019).

25. Guélou, G. et al. Role of excess tellurium on the electrical and thermal properties in Te-doped paracostibite. *J. Mater. Chem. C* **8**, 1811-1818 (2020).

26. Deng, T. et al. Thermoelectric properties of n-type Cu_4_Sn_7_S_16_-based compounds. *RSC Adv.* **9**, 7826-7832 (2019).

27. Liu, J. et al*.* Enhanced thermoelectric performance in ductile Ag_2_S-based materials via doping iodine. *Appl. Phys. Lett.* **119**, 121905 (2021).

28. Ge, B. et al. Enhanced thermoelectric performance of N-type eco-friendly material Cu_1-x_Ag_x_FeS_2_ (x=0–0.14) via bandgap tuning. *J Alloys Compd.* **809**, 151717 (2019).

29. Bourgès, C. et al. Role of cobalt for titanium substitution on the thermoelectric properties of the thiospinel CuTi_2_S_4_. *J Alloys Compd.* **781**, 1169-1174 (2019).

30. Rathore, E. et al*.* Origin of ultralow thermal conductivity in n-type cubic bulk AgBiS_2_: Soft Ag vibrations and local structural distortion induced by the Bi 6s2 Lone Pair. *Chem. Mater.* **31**, 2106-2113 (2019).

31. Hu, X. et al. Thermoelectric transport properties of n-type tin sulfide. *Scripta Mater.* **170**, 99-105 (2019).

32. Shen, X. et al. High thermoelectric performance in sulfide-type argyrodites compound Ag_8_Sn(S_1−x_Se_x_)_6_ enabled by ultralow lattice thermal conductivity and extended cubic phase regime. *Adv. Funct. Mater.* **30**, 2000526 (2020).
